# Is the incidence of survival in interior Pleistocene refugia (nunataks) underestimated? Phylogeography of the high mountain plant *Androsace alpina* (Primulaceae) in the European Alps revisited

**DOI:** 10.1002/ece3.5037

**Published:** 2019-03-07

**Authors:** Peter Schönswetter, Gerald M. Schneeweiss

**Affiliations:** ^1^ Institute of Botany University of Innsbruck Innsbruck Austria; ^2^ Department of Botany and Biodiversity Research University of Vienna Vienna Austria

**Keywords:** Alps, Androsace, high mountain plants, nunataks, peripheral refugia, Pleistocene refugia

## Abstract

Temperate mountain ranges such as the European Alps have been strongly affected by the Pleistocene glaciations. Glacial advances forced biota into refugia, which were situated either at the periphery of mountain ranges or in their interior. Whereas in the Alps peripheral refugia have been repeatedly and congruently identified, support for the latter scenario, termed “nunatak hypothesis,” is still limited and no general pattern is recognizable yet. Here, we test the hypothesis of nunatak survival for species growing in the high alpine to subnival zones on siliceous substrate using the cushion plant *Androsace alpina* (Primulaceae), endemic to the European Alps, as our model species. To this end, we analyzed AFLP and plastid DNA sequence data obtained from a dense and range‐wide sampling. Both AFLPs and plastid sequence data identified the southwestern‐most population as the most divergent one. AFLP data did not allow for discrimination of interior and peripheral populations, but rather identified two to three longitudinally separated major gene pools. In contrast, in the eastern half of the Alps several plastid haplotypes of regional or local distribution in interior ranges—the Alpine periphery mostly harbored a widespread haplotype—were indicative for the presence of interior refugia. Together with evidence from other Alpine plant species, this study shows that in the eastern Alps silicicolous species of open habitats in the alpine and subnival zone survived, also or exclusively so, in interior refugia. As the corresponding genetic structure may be lost in mostly nuclear‐derived, rapidly homogenizing marker systems such as AFLPs or RAD sequencing tags, markers not prone to homogenization, as is the case for plastid sequences (Sanger‐sequenced or extracted from an NGS data set) will continue to be important for detecting older, biogeographically relevant patterns.

## 1 INTRODUCTION

Biota of temperate and northern regions have been strongly affected by Pleistocene climate oscillations. Two independent sets of hypotheses addressing range changes associated with these climatic oscillations have been proposed. One is concerned with whether cold‐adapted species had larger distributions during cold periods, due to expansion into suitable peripheral and lowland regions (interglacial contraction hypothesis; Hewitt, 2004; Stewart, Lister, Barnes, & Dalén, 2010; Espíndola et al., 2012; Theodoridis et al., 2017), or during interglacial periods, when formerly glaciated areas became habitable (interglacial expansion hypothesis; Birks, 2008; Stewart et al., 2010). The second set of hypotheses, which is the focus of this study, is concerned with whether persistence in strongly glaciated areas was possible exclusively in their ice‐free or only weakly glaciated periphery (tabula rasa hypothesis), which may also include areas in lowlands, or also on unglaciated peaks (so‐called nunataks) within the ice shield (nunatak hypothesis; Brochmann, Gabrielsen, Nordal, Landvik, & Elven, 2003; Schneeweiss & Schönswetter, 2011). The different geographic settings of peripheral versus nunatak refugia and the resulting demographic differences with respect to, for instance, population size do not only affect current genetic diversity of a species, but also the extent of colonization of formerly glaciated areas (Willi, van Buskirk, & Hoffmann, 2006). Whereas peripheral refugia have been repeatedly and congruently identified (Allen, Marr, McCormick, & Hebda, 2012; Schönswetter, Stehlik, Holderegger, & Tribsch, 2005; Soltis, Morris, McLachlan, Manos, & Soltis, 2006; Walker, Stockman, Marek, & Bond, 2009), support for nunatak survival is more limited (Escobar García et al., 2012; Stehlik, Blattner, Holderegger, & Bachmann, 2002; Wachter et al., 2016; Westergaard et al., 2011, in press) and no general pattern is recognizable yet.

A geographic model system to test the tabula rasa and nunatak hypotheses are the European Alps, hereinafter simply referred to as the Alps. During glacials, the Alps were nearly entirely covered by ice (Ehlers & Gibbard, 2004) with numerous nunataks within the ice sheet and larger unglaciated areas at the Alpine periphery, most prominently in the southwestern, southern, and eastern Alps (Jäckli, 1970; Nagl, 1972; Schönswetter et al., 2005; Van Husen, 1997). The latter's eminent role as Pleistocene refugia is supported by both biogeographic and molecular data (Tribsch & Schönswetter, 2003; Tribsch, 2004; Schönswetter et al., 2005). In contrast, molecular evidence for nunatak refugia, although already postulated by early Alpine biogeographers (Brockmann‐Jerosch & Brockmann‐Jerosch, 1926), remains limited (Bettin, Cornejo, Edwards, & Holderegger, 2007; Escobar García et al., 2012; Stehlik et al., 2002; Wachter et al., 2016). A potential reason for this is that genetic signatures of nunatak survival may be lost in mostly nuclear‐derived marker systems such as AFLPs or RADseq due to genetic swamping by (re‐)colonizers (Todesco et al., 2016). This is in line with the observation that evidence for nunatak survival of Alpine plants has so far come almost exclusively from plastid markers (e.g., Bettin et al., 2007; Escobar García et al., 2012; Stehlik et al., 2002). Introgression from local nunatak populations into invading populations is expected to be particularly strong in markers experiencing reduced gene flow (Currat, Ruedi, Petit, & Excoffier, 2008) as is the case for plastid markers, which lack recombination and are largely uniparentally inherited in angiosperms (Bock, 2007). Consequently, distinct haplotypes, which are expected to evolve after longer periods of geographic isolation on nunataks, can be retained even in the face of hybridization.

**Figure 1:**
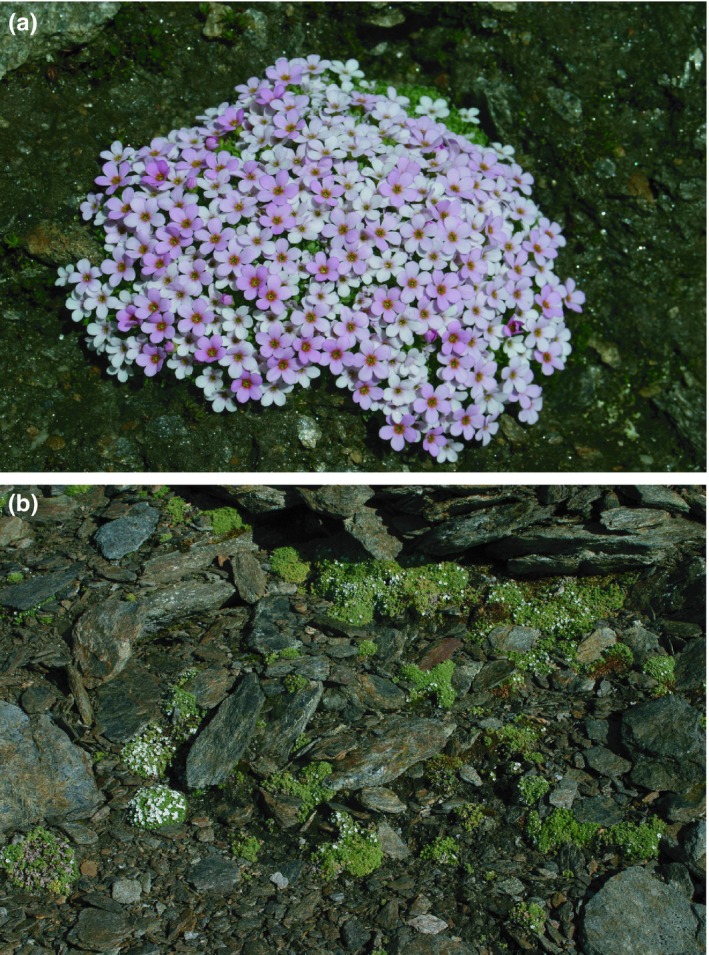
(a) Habit and (b) habitat of the study species *Androsace alpina*

Here, we test the hypothesis of nunatak survival using *Androsace alpina* (Primulaceae; Figure 1a), endemic to the Alps, as our model species. This species is restricted to alpine and subnival zones (Lüdi, 1927), where it grows in fell‐fields, on moraines or rocks (Figure 1b), and as such is expected to have been able to survive (also) on nunataks. Although already studied previously (Schönswetter, Tribsch, & Niklfeld, 2003), the former study only used AFLP data and may, therefore, be biased against identifying interior refugia. Analyzing AFLP and plastid DNA sequence data obtained from a dense and range‐wide sampling, we want to identify the locations of putative refugia of *A. alpina*. Thus, we can assess whether any of the two marker systems commonly used in plant phylogeography, plastid sequences and AFLP data, are biased against identifying interior refugia.

## 2 MATERIALS AND METHODS

**Figure 2:**
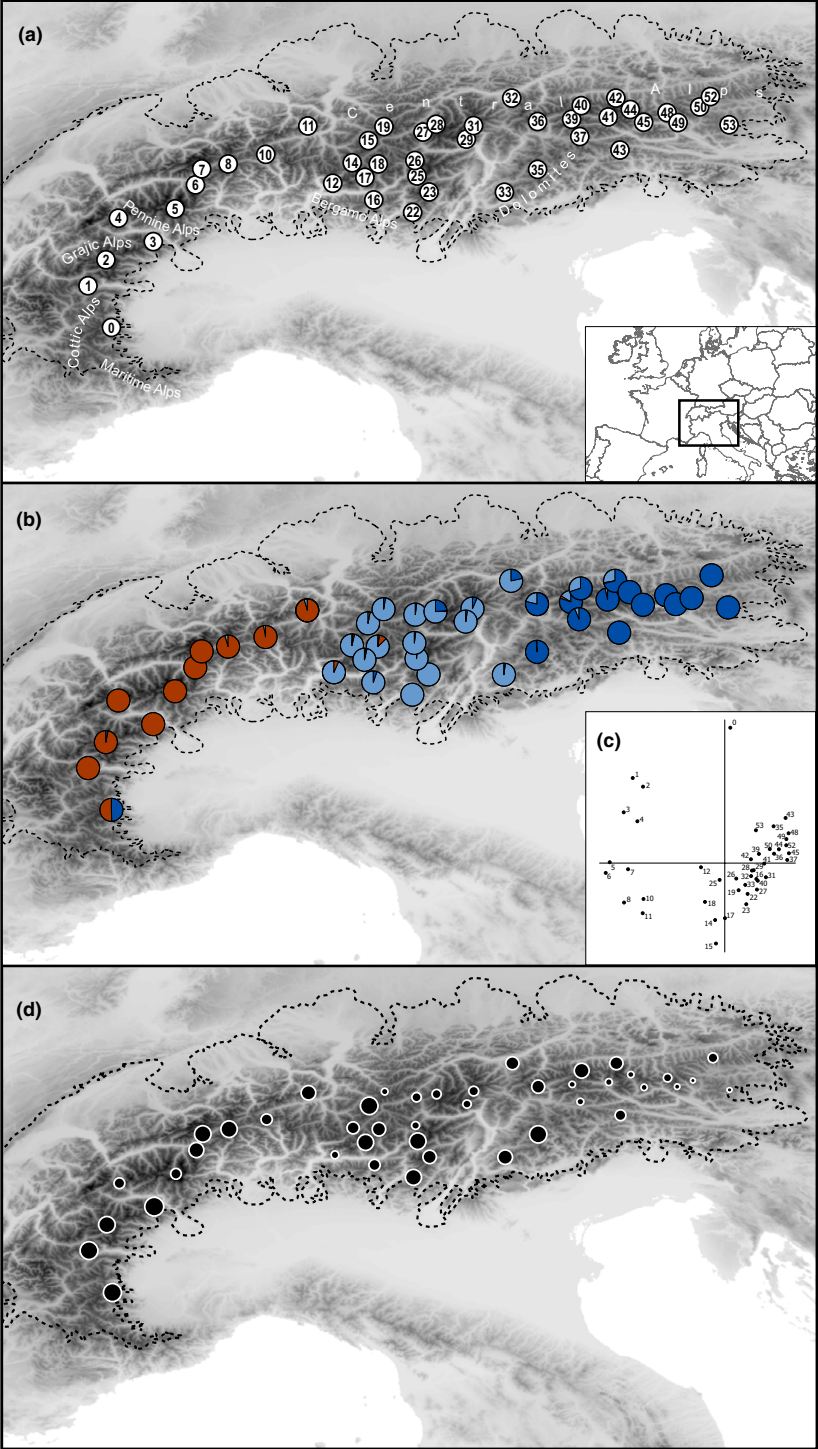
Distribution, sampled populations, and genetic structure of *Androsace alpina* as well as maximum extent of the Alpine ice sheet at the Last Glacial Maximum (dashed line). (a) Sampled populations (see Supporting Information Table S1 for further information); toponyms used in the text are indicated. (b, c) Genetic structure derived from analyses of AFLP markers using (b) the Bayesian clustering approach of STRUCTURE (with the number of clusters, *K*, being 3) or (c) a principal co‐ordinate analysis at the population level. (d) Within‐population rarity of AFLP markers (frequency‐down‐weighted marker values), its magnitude being proportional to dot size

Plants were sampled from 44 populations covering the entire distribution range of this species (Figure 2a) except for the southernmost ones (Maritime Alps, Italy/France). Compared to the previous study of Schönswetter, Tribsch, & Niklfeld (2003), the population‐level sampling has been thinned in the eastern Alps, but extended southwards in the southwestern Alps. For the re‐analyzed populations, numbering corresponds to the previous study and is, therefore, not consecutive (Schönswetter, Tribsch, & Niklfeld, 2003; Supporting Information Table S1). For AFLP data, we included 2–5 (median 4) individuals per population (totaling 159 individuals); for plastid DNA sequencing, we included 2–5 (median 3) individuals per population (totaling 131 individuals). Leaf material was collected and immediately stored in silica gel. Voucher specimens are deposited at the University of Vienna, Austria (WU, voucher details in Supporting Information Table S1).

Total genomic DNA was extracted from dried tissue (c. 10 mg) with the DNeasy 96 plant mini kit (Qiagen, Hilden, Germany) following the manufacturer's protocol. The AFLP procedure was carried out as described in Schönswetter, Solstad, Escobar García, and Elven (2009). We used the same primer combinations for the selective PCR (fluorescent dye in brackets) as in our previous study (Schönswetter, Tribsch, & Niklfeld, 2003), that is, *Eco*RI (6‐Fam)‐ATC/*Mse*I‐CTG, *Eco*RI (VIC)‐ATG/*Mse*I‐CTT, and *Eco*RI (NED)‐AGG/*Mse*I‐CTG. 5 μl of each of the differently labeled selective PCR products were purified using Sephadex G‐50 Fine (GE Healthcare Bio‐Sciences, Uppsala, Sweden) applied to a Multi Screen‐HV plate (Millipore, Molsheim, France). 1.2 μl of the elution product was mixed with 10 μl formamide (Applied Biosystems, Foster City, USA) and 0.1 μl GeneScan 500 ROX (Applied Biosystems), the internal size standard, and run on an ABI 3130x automated capillary sequencer (Applied Biosystems). Raw AFLP data were aligned with the internal size standard using ABI prism genescan 3.7.1 (Applied Biosystems) and imported into genographer 1.6.0 (version no longer available) for scoring. The error rate (Bonin et al., 2004) was calculated based on eight replicated individuals as the ratio of mismatches (scoring of 0 vs. 1) over matches (1 vs. 1) in AFLP profiles of replicated individuals. Fragments with single presences or absences were excluded.

The three plastid regions *ccmp3f*–*trnR*,* rpl20–*5′‐*rps12,* and *trnS*_(UGA)_–*trnfM*_(CAU)_, successfully employed for intraspecific comparisons in other *Androsace* species (Dixon, Schönswetter, & Schneeweiss, 2007, 2008; Dixon, Schönswetter, Suda, Wiedermann, & Schneeweiss, 2009; Dixon, Schönswetter, Vargas, Ertl, & Schneeweiss, 2009; Schneeweiss & Schönswetter, 2010; Schneeweiss, Winkler, & Schönswetter, 2017), were sequenced as described in Dixon, Schönswetter, Suda, et al. (2009) and Schneeweiss et al. (2017). Briefly, the three regions were amplified using standard chemistry with primers *ccmp3f* (Weising & Gardner, 1999) and *trnR* (Dumolin‐Lapegue, Pemonge, & Petit, 1997); *rpl20* and 5′‐*rps12* (both Hamilton, 1999); and *trnS*(UGA) and *trnfM*(CAU) (both Demesure, Sodzi, & Petit, 1995) with the following PCR conditions: 30 s at 96°C; 35 cycles of 5 min at 94°C, 45 s at 48°C, and 10 min at 68°C; 10 min at 68°C. After cleaning the PCR products with Exonuclease I and Calf Intestine Alkaline Phosphatase (Fermentas, St. Leon‐Rot, Germany), cycle sequencing using BigDye Terminator chemistry (Applied Biosystems) followed by electrophoresis with an ABI 3130x capillary sequencer (Applied Biosystems, Foster City, USA) was conducted.

Population structure was inferred using a Bayesian clustering approach developed for dominant markers (Falush, Stephens, & Pritchard, 2007; Pritchard, Stephens, & Donnelly, 2000) as implemented in structure 2.2 run at the Bioportal of the University of Oslo (http://www.bioportal.uio.no/). We used an admixture model with uncorrelated allele frequencies and recessive alleles. Ten replicate runs for each *K* (number of groups) ranging from 1 to 10 were calculated using a burn‐in of 10^5^ iterations followed by 10^6^ additional MCMC iterations. The optimal number of groups was identified using DeltaK (Evanno, Regnaut, & Goudet, 2005) implemented in structureharvester web 0.6.94 (Earl & vonHoldt, 2012). A principal co‐ordinate analysis at the population level (i.e., as done in the previous study of Schönswetter, Tribsch, & Niklfeld, 2003) was computed using FAMD 1.31 (Schlüter & Harris, 2006). Population distances were calculated using chord distances for many loci (Takezaki & Nei, 1996) with null allele frequencies estimated using a nonuniform prior derived from among‐population information (Zhivotovsky, 1999). In order to quantify the genetic “uniqueness” of populations, frequency down‐weighed marker values (DW; Schönswetter & Tribsch, 2005) were calculated for each population (“rarity 1”) with the R‐script AFLPdat (Ehrich, 2006).

DNA sequences were edited with seqman II 5.05 (DNAStar Inc., Madison, WI, USA) and aligned manually using bioedit 7.0.4.1 (Hall, 1999). Prior to all analyses, an inversion in the *ccmp3f*–*trnR* region, present in nearly 40% of the samples, was manually reversed, as it would introduce substitutional mutations, which in fact are the result of a structural mutation (Löhne & Borsch, 2005). A haplotype network was constructed using statistical parsimony as implemented in TCS 1.21 (Clement, Posada, & Crandall, 2000). Since gaps were treated as fifth character state, insertions/deletions of motifs of more than 1 bp and the inversion in the *ccmp3f*–*trnR* region were re‐coded as single characters by reducing them to single base pair columns.

## 3 RESULTS

**Figure 3:**
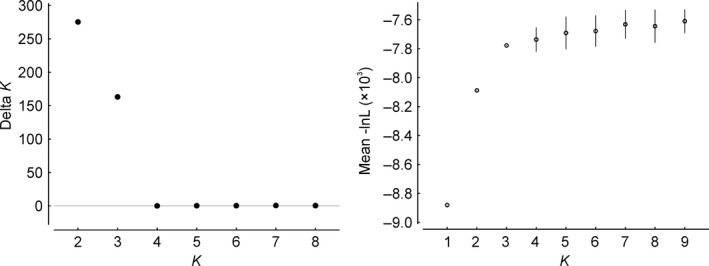
Delta *K* values (left) and ‐ln likelihood (mean and standard deviation; right) derived from analyses of AFLP data using structure for *Androsace alpina*

**Figure 4:**
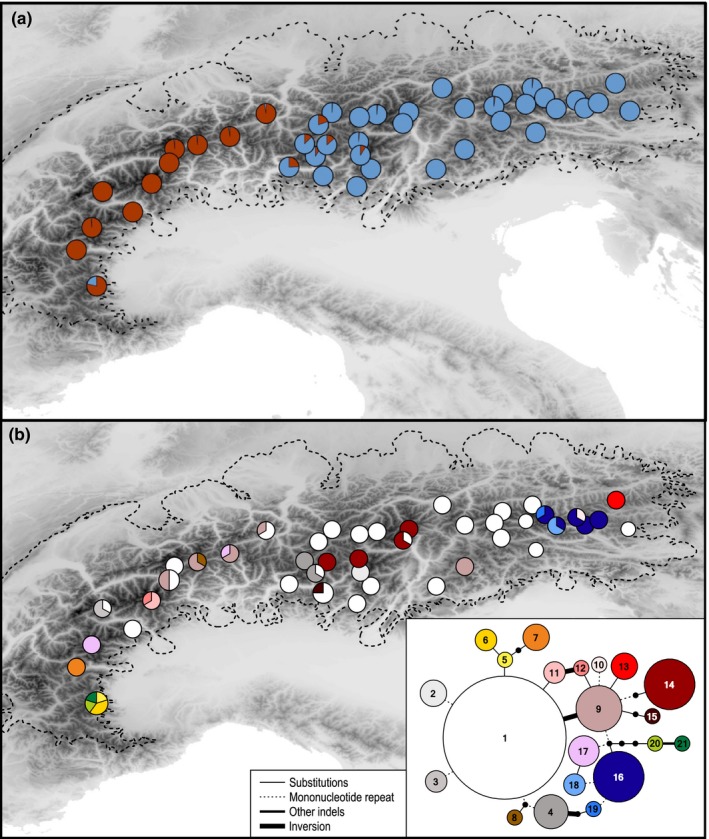
Genetic structure of *Androsace alpina* as well as maximum extent of the Alpine ice sheet at the Last Glacial Maximum (dashed line). (a) Genetic structure derived from analyses of AFLP markers using the Bayesian clustering approach of STRUCTURE with the number of clusters, *K*, being 2. (b) Geographic distribution of the 21 chloroplast DNA haplotypes and, as insert, their relationships visualized as parsimony network, where lines correspond to mutational steps

We scored 165 AFLP fragments in 159 individuals. The error rate (Bonin et al., 2004) was 2.45%. DeltaK identified *K *=* *2 as the optimal number of groups (Figure 3). These groups were separated by longitudinal breaks, one cluster (hereinafter termed Western Cluster) comprising pops. 0–11 from the western Alps, the second (hereinafter termed Eastern Cluster) comprising pops. 12–53 from the eastern Alps (Figure 4a). The southernmost population from the Western Cluster (pop. 0) and western populations from the Eastern Cluster (e.g., pops. 12 and 15) showed admixture, the minority cluster never exceeding 25%. If taking the likelihood distribution over different values of *K* into account, *K *=* *3 was suggested by a stable likelihood maximum (Figure 3). Accordingly, the Eastern Cluster was further divided longitudinally (hereinafter termed Eastern Cluster 1 and Eastern Cluster 2 corresponding to pops. 12–33 and pops. 35–53, respectively), roughly along the Adige valley in northern Italy, with admixture (the minority cluster reaching maximally 30%) in some populations close to the contact zone (Figure 2b). The Western Cluster remained unaffected with the exception of its southernmost population (pop. 0) that showed a nearly 1:1 admixture between the Western Cluster and the Eastern Cluster 2 (Figure 2b).

A principal co‐ordinate analysis (Figure 2c) separated the western populations from the eastern populations along the first axis (27.87%). The western populations were separated along the second axis (13.58%) into three groups arranged latitudinally (pop. 0; pops. 1–4; pops. 5–11). Within the eastern populations, no separation into two groups as identified by structure at *K *=* *3 was evident. The DW varied strongly and ranged from 0.74 in population 53 to 2.23 in population 3 (Figure 2d, Supporting Information Table S1).

Based on plastid sequence data, 21 haplotypes (14 if disregarding mononucleotide repeats) were identified within the investigated species (Figure 4b). The most common and widespread haplotype (HT1) together with rarer haplotypes differing from it only by the number of base pairs in mononucleotide repeats and/or the inversion in *ccmp3f*–*trnR* (HT2, HT3, HT4, HT9, HT10, HT17) jointly were found over the entire distribution range except for the southern southwestern Alps (Figure 4b). The remaining haplotypes, differing from HT1 by at least one nucleotide substitution, were geographically restricted to one or a few populations and thus also to single AFLP clusters. These included several unique haplotypes (HT5–HT7, HT20, HT21) restricted to the southern populations (pops. 0–1) in the southwestern Alps; several unique haplotypes (HT16 as well as HT18 and HT19 differing from it only by the number of base pairs in mononucleotide repeats) restricted to some of the easternmost populations (pops. 44–50); a unique haplotype (HT14) restricted to four populations (pops. 18, 26, 29, 31) in the Central Alps; two unique haplotypes (HT11 and HT12) restricted to a single population in the western Alps (pop. 5 from the Pennine Alps); a unique haplotype (HT13) restricted to the northeasternmost population (pop. 52); and two unique haplotypes (HT8 and HT15) being found in single individuals of single populations otherwise harboring HT1 from the Swiss Alps (pop. 8) and from the southern Alps (pop. 16 from the Bergamo Alps).

## 4 DISCUSSION

In a previous study using AFLP data, Schönswetter, Tribsch, & Niklfeld (2003) identified four groups within *A. alpina*. These are, however, only partially recovered by our structure analyses (Figures 2b and 4a). Specifically, their southwestern and western groups, SW and W (pops 1–4 and pops. 5–11, respectively; pop. 0 was not studied by Schönswetter, Tribsch, & Niklfeld, 2003), are united in the Western Cluster (irrespective of the number of groups, *K *=* *2 or *K *=* *3), whereas their eastern groups, E1 and E2 (pops. 12–18 & 20–26 & 33–34 and pops. 19 & 27–32 & 35–53, respectively), correspond to Eastern Cluster 1 and Cluster 2, respectively, albeit with an eastward shifted boundary, as pops. 19 and 27–32 grouped into E2 by Schönswetter, Tribsch, & Niklfeld (2003) are recovered to belong to the Eastern Cluster 1 from the structure analysis (Figure 2b). These discrepancies likely are due to the use of different methods to delimit groups, that is, principal co‐ordinate analysis, as used by Schönswetter, Tribsch, & Niklfeld (2003), versus structure, as used here. In fact, PCoA from the new data agrees with the previous results (Schönswetter, Tribsch, & Niklfeld, 2003) concerning a strong differentiation within the Western Cluster. No separation into two groups is, however, detected within the Eastern Cluster (Figure 2c), but such a separation was considered ambiguous already in the original study (Schönswetter, Tribsch, & Niklfeld, 2003). Taken together, both previous and new AFLP data are consistent with the hypothesis of the survival of *A. alpina* in three peripheral refugia (from west to east: Cottic Alps; Grajic and Pennine Alps; Bergamo Alps to southern Dolomites) as suggested previously (Schönswetter, Tribsch, & Niklfeld, 2003) and in accordance with patterns found in numerous silicicolous high‐elevation species from the Alps (Schönswetter et al., 2005).

Taking patterns of haplotype divergence and distribution into account, the inference of putative refugia is, however, considerably modified. The three peripheral refugia suggested by AFLP data are supported also by unique haplotypes (Figure 4b). This is particularly pronounced for the southwestern refugium, which harbors five unique haplotypes (four found in the southernmost population pop. 0) what may indicate the presence of distinct microrefugia (Patsiou, Conti, Zimmermann, Theodoridis, & Randin, 2014). The admixture of the southernmost population 0 inferred from the structure analysis (Figure 2b) is likely artifactual. It is known for this software that small, divergent groups—such as this particular population, which forms the sister to all other populations in a neighbor‐joining analysis (not shown)—tend to be resolved as admixed instead of forming separate gene pools (Lawson, van Dorp, & Falush, 2018). For the two peripheral refugia further east support from plastid data is less pronounced. Specifically, two unique haplotypes are found in a single population, pop. 8, from the Pennine Alps and one unique haplotype is present in a single individual from a single population, pop. 16, from the Bergamo Alps. In addition to those three areas, two more are characterized by unique haplotypes (Figure 4b). One is in the western Central Alps (pops. 18, 26, 29, 31), thus being in the same region as the interior refugium suggested for *Senecio carniolicus* s. l. (Escobar García et al., 2012), the second is in the easternmost Central Alps (pops. 44–50), thus encompassing a peripheral refugium identified for several species, including *Androsace wulfeniana* (Primulaceae) and *Saponaria pumila* (Caryophyllaceae; Tribsch, Schönswetter, & Stuessy, 2002; Schönswetter, Tribsch, Schneeweiss, & Niklfeld, 2003).

While not contradicting groups identified by structure analysis of the AFLP data, plastid sequence data allow more refined inferences on Pleistocene refugia. For the Eastern Cluster 1, plastid data identified both peripheral and interior refugia rather than just a peripheral one (Figures 2b and 4b). For the Eastern Cluster 2, plastid data identified a refugium (Figures 2b and 4b), whereas in the previous study of Schönswetter, Tribsch, & Niklfeld (2003) the weak differentiation, together with patterns of diversity and genetic correlations among populations, was interpreted as the result of recent eastward leading edge migration rather than evidence for a separate refugium. Interestingly, the distribution of rare AFLP markers (DW; Figure 2d) does not support a refugium within the Eastern Cluster 2; populations with regionally endemic plastid haplotypes are among the ones with the lowest DW values range‐wide (Supporting Information Table S1). The failure to detect putative refugia by AFLP data likely is data‐type inherent, because the mostly nuclear‐derived, rapidly homogenizing AFLPs are prone to loose signal for in situ survival, if immigrant genotypes swamp resident genotypes (Gabrielsen, Bachmann, Jakobsen, & Brochmann, 1997; Todesco et al., 2016). Swamping by immigrants is likely for the eastern Alps (Eastern Cluster 2), where AFLP data, as mentioned previously, do support an eastward colonization (Schönswetter, Tribsch, & Niklfeld, 2003). Assuming that nunatak populations were small and that colonization progressed mainly from the periphery toward the center following the retreating ice shield, nunatak populations are expected to have been particularly prone to genetic swamping, as evidently has been the case for the interior refugium within Eastern Cluster 1. Major postglacial range shifts are in line not only with the weak phylogeographic structure of *A. alpina* compared to other silicicolous species (Schönswetter, Tribsch, & Niklfeld, 2003), but also with the wide occurrence of a few and, based on their interior position in the haplotype network, probably ancestral haplotypes (e.g., HT1 and HT9).

## 5 CONCLUSION

Phenetic and model‐based phylogeographic analyses of AFLP data return results that, although differing in details, generally yield similar conclusions. Fundamentally different patterns, however, may emerge when combining biparentally inherited, mostly nuclear‐derived, rapidly homogenizing marker systems such as AFLPs or RADseq with mostly uniparentally inherited markers not prone to homogenization, as is the case for plastid or mitochondrial sequences (Wachter et al., 2012; this study). In the present study, the sole use of AFLPs allows only the current relationships determined by massive gene flow over wide distances to be recovered, whereas plastid sequences can identify traces of long‐term survival in interior parts of the Alps, which were the focus of our study. Organellar sequences are no panacea, as even in extreme high‐elevation species such as *Ranunculus glacialis* (Ranunculaceae), where nunatak survival appears equally likely as in *A. alpina*, plastid sequences may fail to provide support for the nunatak hypothesis (Ronikier, Schneeweiss, & Schönswetter, 2012). Likewise, other characteristics of AFLPs, such as frequencies of rare fragments (DW values: Schönswetter & Tribsch, 2005), may provide evidence for nunatak survival (Escobar García et al., 2012), but, as shown by the present study, this is not necessarily the case. We are thus confident that, even in the face of unprecedentedly well‐supported RADseq phylogenies—which like AFLPs may be biased toward reflecting younger history related to, for instance, the current landscape—plastid and mitochondrial data, which are expected to easily introgress into invading, that is, (re‐)colonizing, populations (Currat et al., 2008), will continue to be crucial for detecting older, biogeographically relevant patterns.

## CONFLICT OF INTERESTS

The authors declare that the research was conducted in the absence of any commercial or financial relationships that could be construed as potential competing interests.

## AUTHOR CONTRIBUTIONS

PS and GMS designed the study, gathered and analyzed the data, and prepared the manuscript.

## Data Availability

DNA sequence data are available under GenBank accession numbers MK001843–MK002235. Aligned plastid DNA sequences and the AFLP data matrix are available in dryad under https://doi.org/10.5061/dryad.ts73227 (will be provided upon acceptance).
