# Supplementary material for: Is the incidence of survival in interior Pleistocene refugia (nunataks) underestimated? Phylogeography of the high mountain plant Androsace alpina (Primulaceae) in the European Alps revisited
Source: Ecol Evol. 2019 Mar 7;9(7):4078–86. doi: 10.1002/ece3.5037 (PMC6468090; doi:10.1002/ece3.5037)
Supplement: Supplementary file 1 [file ECE3-9-4078-s001.docx]

**Table S1** Population number, location name, geographic coordinates, number of analysed individuals, frequency down-weighed marker values (DW), plastid haplotypes, and GenBank accession numbers for the investigated populations of *Androsace alpina*.

| Pop. no. | Location | Geographic coordinates^a^ | Number of analysed individuals^b^ | DW | Plastid haplotype^c^ | GenBank accession numbers^d^ |
| --- | --- | --- | --- | --- | --- | --- |
| 0 | Monte Viso | 7.09°/44.67° | 4 / 5 | 1.70 | 5^1^, 6^2^, 20^1^, 21^1^ | MK002166–MK002170; MK001904–MK001908; MK002035–MK002039 |
| 1 | Col Sommelier | 6.83°/45.12° | 4 / 3 | 1.89 | 7 | MK002139–MK002141; MK001877–MK001879; MK002008–MK002010 |
| 2 | Col de l'Iseran | 7.02°/45.42° | 4 / 3 | 1.34 | 17 | MK002200–MK002202; MK001938–MK001940; MK002069–MK002071 |
| 3 | Champorcher | 7.55°/45.62° | 2 / 3 | 2.23 | 1 | MK002206–MK002208; MK001944–MK001946; MK002075–MK002077 |
| 4 | Col Gr. St. Bernard | 7.17°/45.87° | 4 / 3 | 0.70 | 1^1^, 3^2^ | MK002203–MK002205; MK001941–MK001943; MK002072–MK002074 |
| 5 | Gornergrat | 7.80°/45.98° | 4 / 3 | 0.72 | 11^2^, 12^1^ | MK002219–MK002221; MK001957–MK001959; MK002088–MK002090 |
| 6 | Simplon pass | 8.02°/46.23° | 4 / 4 | 1.01 | 1^2^, 9^2^ | MK002209–MK002212; MK001947–MK001950; MK002078–MK002081 |
| 7 | Eggishorn | 8.08°/46.42° | 3 / 3 | 1.33 | 1 | MK002142–MK002144; MK001880–MK001882; MK002011–MK002013 |
| 8 | Nufenen pass | 8.38°/46.47° | 4 / 3 | 1.31 | 8^1^, 9^2^ | MK002145–MK002147; MK001883–MK001885; MK002014–MK002016 |
| 10 | Passo Lucomagno | 8.80°/46.58° | 4 / 3 | 0.75 | 9^2^, 17^1^ | MK002213–MK002215; MK001951–MK001953; MK002082–MK002084 |
| 11 | Cassonsgrat | 9.27°/46.87° | 5 / 3 | 0.89 | 1^2^, 9^1^ | MK002216–MK002218; MK001954–MK001956; MK002085–MK002087 |
| 12 | Monte Spluga | 9.55°/46.18° | 4 / 3 | 0.58 | 1 | MK002108–MK002110; MK001846–MK001848; MK001977–MK001979 |
| 14 | Piz Julier | 9.75°/46.48° | 4 / 3 | 0.78 | 4 | MK002133–MK002135; MK001871–MK001873; MK002002–MK002004 |
| 15 | Schwarzhorn | 9.93°/46.73° | 3 / 3 | 1.46 | 1 | MK002127–MK002129; MK001865–MK001867; MK001996–MK001998 |
| 16 | Pizzo di Coca | 10.00°/46.07° | 4 / 4 | 0.76 | 1^3^, 15^1^ | MK002111–MK002114; MK001849–MK001852; MK001980–MK001983 |
| 17 | Bocchetta Forbici | 9.90°/46.32° | 3 / 3 | 1.18 | 1^1^, 4^2^ | MK002105–MK002107; MK001843–MK001845; MK001974–MK001976 |
| 18 | Monte Breva | 10.05°/46.47° | 4 / 3 | 0.85 | 14 | MK002124–MK002126; MK001862–MK001864; MK001993–MK001995 |
| 19 | Hohes Rad | 10.10°/46.88° | 4 / 3 | 0.56 | 1 | MK002160–MK002162; MK001898–MK001900; MK002029–MK002031 |
| 22 | Passo Crocedomini | 10.43°/45.93° | 4 / 3 | 1.11 | 1 | MK002118–MK002120; MK001856–MK001858; MK001987–MK001989 |
| 23 | Val Folgorida | 10.61°/46.17° | 4 / 3 | 0.83 | 1 | MK002121–MK002123; MK001859–MK001861; MK001990–MK001992 |
| 25 | Passo di Gavia | 10.47°/46.33° | 4 / 3 | 1.29 | 2 | MK002115–MK002117; MK001853–MK001855; MK001984–MK001986 |
| 26 | Stilfser Joch | 10.46°/46.52° | 4 / 3 | 0.59 | 14 | MK002171–MK002173; MK001909–MK001911; MK002040–MK002042 |
| 27 | Piz Lad | 10.47°/46.83° | 4 / 3 | 0.64 | 1 | MK002130–MK002132; MK001868–MK001870; MK001999–MK002001 |
| 28 | Weissseejoch | 10.68°/46.87° | 4 / 3 | 0.64 | 1 | MK002163–MK002165; MK001901–MK001903; MK002032–MK002034 |
| 29 | Pfossental | 11.02°/46.75° | 4 / 3 | 0.62 | 1^1^, 14^2^ | MK002233–MK002235; MK001971–MK001973; MK002102–MK002104 |
| 31 | Timmelsjoch | 11.10°/46.90° | 5 / 3 | 0.66 | 14 | MK002174–MK002176; MK001912–MK001914; MK002043–MK002045 |
| 32 | Glungezer | 11.52°/47.20° | 3 / 3 | 0.80 | 1 | MK002157–MK002159; MK001895–MK001897; MK002026–MK002028 |
| 33 | Monte Ziolera | 11.45°/46.17° | 4 / 3 | 0.87 | 1 | MK002148–MK002150; MK001886–MK001888; MK002017–MK002019 |
| 35 | Passo Pordoi | 11.82°/46.42° | 5 / 3 | 1.37 | 9 | MK002136–MK002138; MK001874–MK001876; MK002005–MK002007 |
| 36 | Tristenspitz | 11.82°/46.95° | 2 / 3 | 0.81 | 1 | MK002230–MK002232; MK001968–MK001970; MK002099–MK002101 |
| 37 | Toblacher Pfannhorn | 12.28°/46.78° | 4 / 3 | 0.55 | 1 | MK002191–MK002193; MK001929–MK001931; MK002060–MK002062 |
| 39 | Totenkarspitze | 12.18°/46.97° | 3 / 3 | 0.53 | 1 | MK002227–MK002229; MK001965–MK001967; MK002096–MK002098 |
| 40 | Obersulzbachtal | 12.30°/47.12° | 4 / 3 | 0.98 | 1 | MK002154–MK002156; MK001892–MK001894; MK002023–MK002025 |
| 41 | Kalser Höhe | 12.6°/47.00° | 3 / 2 | 0.57 | 1 | MK002225–MK002226; MK001963–MK001964; MK002094–MK002095 |
| 42 | Kitzsteinhorn | 12.68°/47.20° | 4 / 3 | 0.79 | 1 | MK002151–MK002153; MK001889–MK001891; MK002020–MK002022 |
| 43 | Monte Peralba | 12.72°/46.63° | 4 / 3 | 0.71 | 1 | MK002188–MK002190; MK001926–MK001928; MK002057–MK002059 |
| 44 | Hochtor | 12.83°/47.08° | 2 / 3 | 0.54 | 16^2^, 19^1^ | MK002222–MK002224; MK001960–MK001962; MK002091–MK002093 |
| 45 | Sadnig | 12.98°/46.93° | 3 / 3 | 0.53 | 16^1^, 18^2^ | MK002194–MK002196; MK001932–MK001934; MK002063–MK002065 |
| 48 | Ankogel | 13.25°/47.05° | 4 / 3 | 0.63 | 10^1^, 16^2^ | MK002180–MK002182; MK001918–MK001920; MK002049–MK002051 |
| 49 | Reisseck | 13.36°/46.95° | 3 / 3 | 0.45 | 16 | MK002177–MK002179; MK001915–MK001917; MK002046–MK002048 |
| 50 | Wandspitze | 13.53°/47.02° | 4 / 3 | 0.38 | 16 | MK002197–MK002199; MK001935–MK001937; MK002066–MK002068 |
| 52 | Hochgolling | 13.76°/47.27° | 4 / 3 | 0.66 | 13 | MK002183–MK002185; MK001921–MK001923; MK002052–MK002054 |
| 53 | Bretthöhe | 13.93°/46.91° | 2 / 2 | 0.37 | 1 | MK002186–MK002187; MK001924–MK001925; MK002055–MK002056 |

^a^ longitude [E]/ latitude [N]

^b^ in case of more than one haplotype present per population, the number of individuals carrying the haplotypes is given as superscript number

^c^ AFLPs / plastid data

^d^ given in the order *ccmp3f*–*trnR*; *rpl20–*5’-*rps12*; *trnS*_(UGA)_–*trnfM*_(CAU)_
